# Time course and dose response of alpha tocopherol on oxidative stress in haemodialysis patients

**DOI:** 10.1186/1471-2369-10-32

**Published:** 2009-10-22

**Authors:** Ashleigh Reed, Yeoung Jee Cho, Jeff S Coombes, Robert G Fassett

**Affiliations:** 1 School of Human Movement Studies, The University of Queensland, St. Lucia, Queensland, 4072, Australia; 2 Renal Medicine, Royal Brisbane and Women's Hospital, Brisbane, Queensland, 4029, Australia; 3 The School of Medicine, The University of Queensland, Brisbane, Queensland, 4029, Australia

## Abstract

**Background:**

Oxidative stress is associated with increased cardiovascular morbidity and mortality particularly in patients with end stage kidney disease. Although observational data from the general population has shown dietary antioxidant intake is associated with reduced cardiovascular morbidity and mortality, most clinical intervention trials have failed to support this relationship. This may be a consequence of not using an effective antioxidant dose and/or not investigating patients with elevated oxidative stress. The SPACE study, conducted in haemodialysis patients, reported that 800 IU/day of alpha tocopherol significantly reduced cardiovascular disease endpoints. A recent time course and dose response study conducted in hypercholesterolaemic patients that found 1600 IU/day of alpha tocopherol was an optimal dose. There is no such dose response data available for haemodialysis patients. Therefore the aim of this study is to investigate the effect of different doses of oral alpha tocopherol on oxidative stress in haemodialysis patients with elevated oxidative stress and the time taken to achieve this effect.

**Methods:**

The study will consist of a time-course followed by a dose response study. In the time course study 20 haemodialysis patients with elevated oxidative stress will take either 1600 IU/day natural (RRR) alpha tocopherol for 20 weeks or placebo. Blood will be collected every two weeks and analysed for a marker of oxidative stress (plasma F_2_-isoprostanes) and alpha tocopherol. The optimum time period to significantly decrease plasma F_2_-isoprostanes will be determined from this study. In the dose response study 60 patients will be randomised to receive either placebo, 100, 200, 400, 800 or 1600 IU/day of natural (RRR) alpha tocopherol for a time period determined from the time course study. Blood will be collected at baseline and every two weeks and analysed for plasma F_2_-isoprostanes and alpha tocopherol. It is hypothesised that doses ≥ 800 IU of vitamin E will be required to significantly decrease plasma F_2_-isoprostanes.

**Discussion:**

This study will determine the time and dose required for alpha tocopherol to significantly decrease oxidative stress in haemodialysis patients. Data will be used to plan a large randomised controlled trial to assess the effects of alpha tocopherol on cardiovascular outcomes in haemodialysis patients.

**Trial Registration:**

ACTRN12609000608268

## Background

Oxidative stress is an important contributor to the development of atherosclerosis and hence cardiovascular morbidity and mortality [1]. Observational studies suggest diets high in antioxidants such as alpha tocopherol may be associated with decreased cardiovascular disease [2,3]. However, antioxidant intervention trials have failed to demonstrate cardiovascular mortality benefits [4-11]. This may be due to the type and dose of antioxidant selected and the features of the populations studied. For example, trials have not selected elevated oxidative stress as an inclusion criteria therefore the study participants may not have been the most likely to benefit.

Chronic kidney disease is associated with high levels of oxidative stress, and treatment with haemodialysis can further increase oxidative stress levels, which may contribute to high level of cardiovascular disease in these patients [1,12]. These patients have a 10-20 times higher rate of cardiovascular disease morbidity and mortality compared to healthy people [13,14].

Alpha tocopherol is the main form of vitamin E and this has been the most commonly studied dietary antioxidant supplement in clinical trials. In one of the few trials to demonstrate a beneficial cardiovascular affect, the Secondary Prevention with Antioxidants of Cardiovascular disease in End stage renal disease (SPACE) study assessed the effects of 800 IU of alpha tocopherol on cardiovascular outcomes in 196 haemodialysis patients [15]. In this randomised controlled trial there was a significant reduction in cardiovascular disease endpoints and myocardial infarction in treated patients. However, the design of this study has been criticised, mainly for failure to measure oxidative stress and vitamin E levels in most of the patients [16-18] and because the study had a small sample size with resultant wide confidence intervals for a broad composite endpoint [19,20]. The authors responded but only reported vitamin E and oxidative stress measures in 15 patients from each group [21].

A recognised weakness of many of the large vitamin E cardiovascular trials is a lack of oxidative stress data assessing whether the supplementation was pharmacologically effective. In addition there has been evidence from meta-analyses indicating potential safety concerns with higher doses of alpha tocopherol [22].

Roberts et al (2007) conducted a time course and dose determining study in hypercholesterolemic patients. They reported that 1600 IU of alpha tocopherol was required over 16 weeks to significantly reduce plasma F_2_-isoprostanes, a biomarker of oxidative stress [23]. No such data is available for haemodialysis patients. Hence, this study has been designed using this approach to establish a dose of alpha tocopherol that will achieve a significant reduction in oxidative stress, and to determine how long it would need to be administered for, to achieve this effect. This information will be used to plan a large, randomised controlled trial to assess the effects of alpha tocopherol on cardiovascular outcomes in haemodialysis patients.

## Methods

### Design and Setting

This investigation will involve two sequential components, a time course, followed by a dose response study. The studies will be conducted at the Renal Medicine Department of the Royal Brisbane and Women's Hospital, Brisbane, Australia, which services a population of approximately 1.2 million people.

### Ethical Considerations

The Royal Brisbane and Women's Hospital Research and Ethics Committee approved the studies. The Ethics Committee will promptly receive all adverse event reports along with a final report.

### Eligibility

For both studies, inclusion criteria are; age > 18 and < 85 years, incident and prevalent dialysis patients, who are undergoing regular three times per week, hospital based haemodialysis and who have elevated oxidative stress. Elevated oxidative stress will be defined as having total plasma F_2_-isoprostane levels > 500 pg/mL. This is based on a study that compared plasma F_2_-isoprostane levels in chronic haemodialysis patients (1662 ± 289 pg/mL) with matched healthy controls (396 ± 18 pg/mL) [24,25] and the Roberts et al. (2007) study that defined elevated oxidative stress as two standard deviations above the mean [23].

Patients will be excluded if they are currently taking alpha tocopherol or have been taking it in the previous three months for at least one week, and if they are unable, or unwilling to give informed consent. In addition, patients who are participating in another investigational study, or are within 30 days of completing an investigational study, or patients who are taking warfarin, desimpramine, chlorpromazine and chloroquine will also be ineligible.

### Identification of Eligible Patients

Eligible patients will be screened from the haemodialysis unit and will receive a copy of the patient information and consent form. An investigator will then explain the study during a clinical consultation. The patient will then be asked to take the information and consent form away with them and make a decision before their next haemodialysis visit. If they agree to participate, they will sign the consent form with an independent person signing as a witness.

### Randomization

An individual not associated with the trial will perform the randomization for both studies using a computer generated random number system.

### Time-course study

Twenty participants will be recruited for the time-course study with ten randomly allocated to a treatment group and ten to a placebo group. Both groups will be required to take two capsules per day (one in the morning and one at night) each containing either a placebo or 800 IU of natural (RRR) alpha tocopherol (total 1600 IU/day) for 20 weeks.

### Dose-response study

Following the time course study, sixty participants will be recruited for the dose-response study with placebo, 100 IU/day, 200 IU/day, 400 IU/day, 800 IU/day and 1600 IU/day natural (RRR) alpha tocopherol groups each consisting of ten people. If patients from the time course study agree to participate in the dose-response study they will undergo a 12-week period (wash out) where they will not be consuming alpha tocopherol. Dose-response study participants will be required to take two capsules per day (one in the morning and one at night) containing either a placebo or alpha tocopherol, to provide the respective IU/day doses.

The placebo will contain the emulsifying agent (wheatgerm oil) used in the alpha tocopherol supplement. Cognis Nutrition & Health Pty Ltd, Sydney, Australia, will supply all capsules.

### Blinding and concealment

All vitamin E and placebo capsules will be identical. The allocation will be provided coded to the person who dispenses the medication thus ensuring the dispenser and patient are blinded to the allocation.

### Adverse event monitoring

A medical co-investigator will assess whether reported adverse events are related to the study medication and will also assess all signs, symptoms and complaints. All adverse events, regardless of relationship to the study, will be recorded and will include; the specific condition or event and direction of change, whether the condition is pre-existing, the dates and times of the occurrence, the severity and significance to study, the action taken and the outcome.

Serious adverse events recorded will include death, in-patient hospitalisation, persistent or significant disability or incapacity, cancer, or be life threatening. Additionally, important medical events that may not result in the above stated outcomes but may require medical or surgical intervention will also be reported.

### Primary Objectives and Outcome Measures

The first primary objective is to determine the time point when oxidative stress is significantly reduced in haemodialysis patients taking 1600 IU/day of alpha tocopherol. Based on the Roberts et al. (2007) study in hypercholesterolemic patients, it is hypothesised that this will occur at 16-weeks [23].

Using this information, the second primary objective is to determine the optimal dose of alpha tocopherol required to reduce oxidative stress in patients undergoing haemodialysis. Based on the SPACE trial, it is hypothesised that ≥ 800 IU of alpha tocopherol will be required to significantly reduce oxidative stress [15].

### Secondary Objectives and Outcome Measures

For both studies, secondary objectives will be to determine the effects of alpha tocopherol on additional measures of oxidative stress and antioxidant status. The additional measures will be plasma protein carbonyls, malondialdehyde and antioxidant enzyme activities of superoxide dismutase, glutathione peroxidase and catalase.

### Visit One (establishment of oxidative stress status)

After obtaining written, signed and witnessed informed consent patients will have blood taken from the dialysis line, at the start of dialysis, to measure total plasma F_2_-isoprostanes to assess their eligibility (> 500 pg/ml) to progress to the next phase of the study.

### Visit two

Once randomized, patients will be booked into a clinic appointment for baseline trial measures. This will occur on a mid-week dialysis day. At the first trial visit, additional data will be obtained from the medical records (medical history, medications) and measures of height, weight and blood pressure will be recorded. Additional blood will be collected pre-dialysis for routine blood chemistry and for measures of plasma F_2_-isoprostanes and alpha tocopherol.

### Time course study

Participants recruited into the time-course study will be required to consume 1600 IU of alpha tocopherol orally each day for 20 weeks or an identical placebo. Blood samples of 8 mL will be taken once every fortnight from the dialysis line, at the start of dialysis, during a normal mid-week dialysis visit and analysed for plasma F_2_-isoprostanes and alpha tocopherol.

### Dose-response study

Participants recruited for the dose-response study will be allocated after randomisation to consume either a placebo, 100 IU, 200 IU 400 IU, 800 IU or 1600 IU of alpha tocopherol orally each day for the duration determined by the time-course study. Blood samples of 8 mL will be taken at the completion of the time period from the dialysis line, at the start of dialysis, during their normal mid-week dialysis visit and analysed for plasma F_2_-isoprostanes and alpha tocopherol

### Adherence to Therapy

Patients will be required to return the container each month with unused capsules inside. Adherence to therapy will be assessed by duplicate table counts of the remaining capsules conducted independently by two study investigators.

### Withdrawal from Study

Patients will be withdrawn from the study at their request, without prejudice, as documented and explained at the time of consenting. Patients who withdraw will be invited to consent to follow-up testing for the remainder of the trial to enable an intention to treat data analysis.

### Blood collection, separation and storage

EDTA blood (approx. 8 mL) will be collected from the sample line shortly after the patient has commenced dialysis. If there is considerable time (> 10 mins) between blood collection and separation then the blood sample will be placed on ice. Centrifugation will be at 600 × g for 10 mins with plasma aliquots then stored at -80°C.

### F_2_-isoprostanes

Total isoprostanes (8-iso-PGF_2α_) will be extracted from plasma according to a modified method of Taylor and colleagues [26]. Samples from the freezer will be thawed on ice then diluted with distilled water and spiked with an internal standard; 8-iso-PGF_2α_-d_4 _(Cayman chemical, Michigan, USA) before methanolic NaOH solution is added. The solution will be vortexed, caped and placed in a water bath at 37°C for 60 min. This is followed by further dilution with distilled water and then acidification to pH 3 with hydrochloric acid before being extracted with hexane. Following gentle rotation and centrifugation at 3000 × *g*, hexane extracts will be removed and discarded. The aqueous layer will then be extracted twice with ethyl acetate, with extracts pooled and dried under nitrogen. Once dry, the samples will be reconstituted with acetonitrile, vortexed and transferred into 300 μL glass inserts and dried ready for derivatisation. The derivatization method will be based on that used by Mori and colleagues [27]. To the dried extract, pentafluorobenzylbromide and 20 μL diisopropylethylamine will be added and incubated at room temperature for 30 min. Following incubation, samples will be further dried under nitrogen to give the F_2_-isoprostane pentafluorobenzyl ester. Samples will then be treated with pyridine, bis(trimethylsilyl)trifluoroacetamide and trimethylchlorosilane and incubated at 45°C for 20 min to yield the trimethylsilylethers. After incubation, hexane will be added to each sample, mixed and placed in the auto sampler. Samples will then be analyzed using a Varian 320 MS/MS with a Varian 450 gas chromatograph using Varian MS Workstation - system control software version 6.9.1 (Varian, Australia). Using a 10 μL Hamilton syringe with 1.2 μL of sample injected for each analysis. The injector will operate at 250°C with a Varian FactorFour Capillary Column - VF - 5 ms 30 m × 0.25 mm ID DF = 0.25 using helium as the carrier gas at a flow rate of 1.0 ml·min^-1 ^with pressure maintained at 13.9 psi. The column oven will start at 160°C held for one minute then increase to 300°C over 7 min then holding for 10 min for an overall run time of 18 min with the peaks eluting around 10.3 min. Running in negative chemical ionization mode the reagent gas will be methane with an ion source pressure of 7.00 Torr and argon as the collision gas at a pressure of 2.00 mTorr. The mass size of our compounds is expected to be 569 and 573 for isoprostanes and the internal standard, respectively.

### Alpha tocopherol

Plasma alpha tocopherol levels will analysed using a modified HPLC method from Katsandinis et al. [28]. Plasma aliquots will be thawed on ice and mixed with ethanol/BHT and vortexed. Hexane will then be added and samples gently mixed for 10 mins before being centrifuged at 4,000 × g for 10 mins. The hexane layer will be removed, dried and reconstituted with mobile phase containing hexane and isopropanol. Vitamin E concentrations will be measured at 295 nm using HPLC (Shimazu) with a LiChrospher C18 column (250 × 4 mm, 5 μm, 1 ml/min flow rate, 9 MPa backpressure).

### Sample Size Calculation

The primary outcome measure for both studies will be the total plasma F_2_-isoprostane levels. For both studies, we will assume that the initial plasma F_2_-isoprostane levels will be 1600 ± 300 pg/mL. This is based on the inclusion criteria requiring patients to have plasma F_2_-isoprostane levels > 500 pg/mL, and also data showing plasma F_2_-isoprostane levels in haemodialysis patients were 1662 ± 289 pg/mL [24,25]. A decrease in plasma F_2_-isoprostane levels to 50% (800 pg/mL) of the initial values will be regarded as significant. To our knowledge, there is little data to support this assumption as no studies have assessed the relationships between plasma F_2 _isoprostanes and long-term health outcomes. Data from the Roberts et al. (2007) study found an approximately 50% reduction in plasma F_2_-isoprostane levels after 16 weeks vitamin E treatment in hypercholesterolemic patients [23]. With these assumptions, accepting their limitations and using a change variance of 350 pg/mL, with alpha = 0.05, beta = (1-0.1 = 0.9) we require five subjects per group. Assuming that five subjects per group will withdraw, die or receive a kidney transplant during the trial we will recruit ten subjects per group for both studies.

### Statistical Analyses

The analysis of both the time-course and dose-ranging study will focus on analysing the changes in the levels of plasma F_2_-isoprostanes and alpha tocopherol, which occur during the intervention. The time-course study will use general linear modelling to analyse the levels of plasma F_2_-isoprostanes at bi-weekly intervals and levels of alpha tocopherol will be compared at baseline and week 20. In the dose-ranging study we will use general linear modelling to compare the changes in plasma F_2_-isoprostane levels between the intervention and placebo group at baseline and at the completion of the study. All statistical analyses will be performed using STATA statistical software (STATA 10; Statistical data analysis; Stata Corp; College Station; Texas, USA).

## Discussion

Despite observational studies suggesting alpha tocopherol may protect from cardiovascular disease, most interventional studies have failed to support this contention. There may be many reasons for this discrepancy including the fact many patients treated in such studies may not have had elevated levels of oxidative stress. In addition, the administered alpha tocopherol may not have lowered oxidative stress. This time course and dose response study of alpha tocopherol in haemodialysis patients will provide data to support further large randomised controlled trials in this area.

## Competing interests

The authors declare that they have no competing interests.

## Authors' contributions

AR, YJC, RGF and JSC are responsible for the design of this trial, and the construction of the protocol. All authors contributed to, and approved the final manuscript.

## Pre-publication history

The pre-publication history for this paper can be accessed here:

http://www.biomedcentral.com/1471-2369/10/32/prepub
